# Technical note: Accelerated nonrigid motion‐compensated isotropic 3D coronary MR angiography

**DOI:** 10.1002/mp.12663

**Published:** 2017-12-12

**Authors:** Teresa Correia, Gastão Cruz, Torben Schneider, René M. Botnar, Claudia Prieto

**Affiliations:** ^1^ Division of Imaging Sciences and Biomedical Engineering King's College London London UK; ^2^ Philips Healthcare Guildford Surrey UK; ^3^ Pontificia Universidad Católica de Chile Escuela de Ingeniería Santiago Chile

**Keywords:** compressed sensing, coronary MRA, image navigator, respiratory motion compensation

## Abstract

**Purpose:**

To develop an accelerated and nonrigid motion‐compensated technique for efficient isotropic 3D whole‐heart coronary magnetic resonance angiography (CMRA) with Cartesian acquisition.

**Methods:**

Highly efficient whole‐heart 3D CMRA was achieved by combining image reconstruction from undersampled data using compressed sensing (CS) with a nonrigid motion compensation framework. Undersampled acquisition was performed using a variable‐density Cartesian trajectory with radial order (VD‐CAPR). Motion correction was performed in two steps: beat‐to‐beat 2D translational correction with motion estimated from interleaved image navigators, and bin‐to‐bin 3D nonrigid correction with motion estimated from respiratory‐resolved images reconstructed from undersampled 3D CMRA data using CS. Nonrigid motion fields were incorporated into an undersampled motion‐compensated reconstruction, which combines CS with the general matrix description formalism. The proposed approach was tested on 10 healthy subjects and compared against a conventional twofold accelerated 5‐mm navigator‐gated and tracked acquisition.

**Results:**

The proposed method achieves isotropic 1.2‐mm Cartesian whole‐heart CMRA in 5 min ± 1 min (~8× acceleration). The proposed approach provides good‐quality images of the left and right coronary arteries, comparable to those of a twofold accelerated navigator‐gated and tracked acquisition, but scan time was up to about four times faster. For both coronaries, no significant differences (*P* > 0.05) in vessel sharpness and length were found between the proposed method and reference scan.

**Conclusion:**

The feasibility of a highly efficient motion‐compensated reconstruction framework for accelerated 3D CMRA has been demonstrated in healthy subjects. Further investigation is required to assess the clinical value of the method.

## 1 Introduction

Coronary artery disease (CAD) remains the leading cause of death worldwide, affecting both men and women in developed and developing countries. The disease occurs when atherosclerotic plaque accumulates in the coronary arteries, thereby obstructing blood flow. Coronary computed tomography angiography (CTA) is an established noninvasive technique for assessing CAD, with a diagnostic accuracy similar to invasive coronary angiography, which is regarded as the “gold standard” for the detection of coronal luminal stenosis.

Coronary magnetic resonance angiography (CMRA) is a promising noninvasive imaging modality for the detection of CAD. Its high soft‐tissue contrast and absence of ionizing radiation is a big advantage over CTA. Moreover, coronary arteries may be visualized without the use of exogenous contrast agents. Additionally, CMRA may provide better diagnosis in patients with heavily calcified plaque in the coronary arteries, which is the main cause of false‐positive readings in CTA and consequent unnecessary referrals for invasive coronary angiography.

Coronary arteries are tortuous structures, small in diameter, and cover a large volume. Therefore, for accurate diagnosis and assessment of stenosis severity, CMRA requires high isotropic spatial resolution and sufficient volumetric coverage to cover the entire coronary artery tree. However, there are major technical challenges associated with this technique, such as image quality degradation due to respiratory motion and long scan times.

Breathing leads to a shift and deformation of the heart mainly in the superior–inferior (SI) direction,^1^ and also to additional 3D affine and nonrigid components that differ strongly between different subjects.^2^, ^3^ The most commonly used motion compensation strategy in whole‐heart CMRA is diaphragmatic one‐dimensional (1D) navigator gating and tracking.^4^ However, this approach only compensates for SI translational motion and its scan efficiency is low, leading to prolonged and unpredictable acquisition times as data outside the gating window are rejected and must be reacquired. Conventionally, acquisition times can be reduced by sacrificing spatial resolution, volumetric coverage or increasing the acceptance window size. Additionally, motion within this window is estimated from the right hemi‐diaphragmatic displacement using a fixed scaling factor of 0.6.^5^ However, this value is not optimal for all subjects and regions of the heart.^6^ Hence, residual motion artifacts may be present in the images. Respiratory 1D self‐navigation techniques have been proposed as an alternative approach to compensate for respiratory motion while also increasing scan efficiency.^7^, ^8^, ^9^, ^10^ These methods estimate the translational displacement of the heart due to respiration in the SI direction directly from the data. However, motion correction is still limited to translation in one dimension and static tissue (e.g., chest wall fat) can lead to motion estimation errors.

Several motion compensation approaches have been recently proposed to achieve high scan efficiency and correct for more complex motion in fully sampled free‐breathing acquisitions, allowing reduced and predictable scan times. These approaches correct for beat‐to‐beat translational motion based on two‐dimensional (2D) or three‐dimensional (3D) image navigators (iNAVs)^11^, ^12^, ^13^, ^14^ or correct for more complex affine motion, estimating motion from iNAVs^15^ or the data itself.^16^, ^17^, ^18^ In the former, low spatial resolution or undersampled iNAVs are acquired at each cardiac cycle. In the latter, iNAVs (or CMRA data) are grouped into multiple respiratory states (or “bins”) over several cardiac cycles, providing higher spatial but lower temporal resolution images, allowing estimation of bin‐to‐bin motion. More complex image‐based motion correction techniques have also been proposed to correct nonrigid motion components that cannot be corrected with an affine model.^19^, ^20^, ^21^, ^22^, ^23^ Recently, a method has been proposed that combines beat‐to‐beat 2D translational correction with bin‐to‐bin 3D nonrigid motion correction.^19^

Another approach used to overcome the prohibitively long acquisition times in whole‐heart CMRA, particularly in the case of isotropic resolution, is to accelerate acquisitions using sub‐Nyquist sampling techniques, such as parallel imaging^24^, ^25^ and compressed sensing (CS).^26^, ^27^ Currently, parallel imaging techniques are widely used in clinical MR, but often limited to a moderate twofold acceleration. CS has attracted considerable attention in recent years as it allows substantial additional acceleration, beyond parallel imaging capabilities. The theory of CS states that if an image is sparse in a transform domain, then it can be accurately reconstructed from incomplete datasets using nonlinear optimization techniques, provided that the undersampling aliasing artifacts are incoherent, i.e., noise‐like.^28^ So far, very few methods that exploit the sparsity or compressibility of images have been combined with translational motion correction or respiratory data binning for additional scan acceleration.^29^, ^30^ However, these methods do not compensate for more complex nonrigid motion.

In this work, we propose a motion‐compensated reconstruction framework for accelerated 3D CMRA, which combines a CS‐based undersampled reconstruction with the nonrigid motion compensation strategy proposed by Cruz et al.,^19^, ^31^ to further accelerate the scan, and thus, enable isotropic Cartesian CMRA acquisitions. Moreover, here we use a variable‐density Cartesian trajectory with radial order (VD‐CAPR) to obtain high‐quality respiratory‐resolved reconstructions from highly undersampled data, essential for accurate estimation of 3D nonrigid motion parameters. This trajectory introduces minimal aliasing artifacts and fully samples the central k‐space region, thereby effectively reducing sensitivity to motion artifacts from residual breathing and cardiac motion. The proposed Accelerated reconstruction with Cartesian‐Ordered acquisition and Motion Correction (ACOMoCo) was tested on 10 healthy subjects and compared against a conventional twofold accelerated navigator‐gated and tracked acquisition.

## 2 Materials and methods

### 2.A. Image acquisition

**Figure 1:**
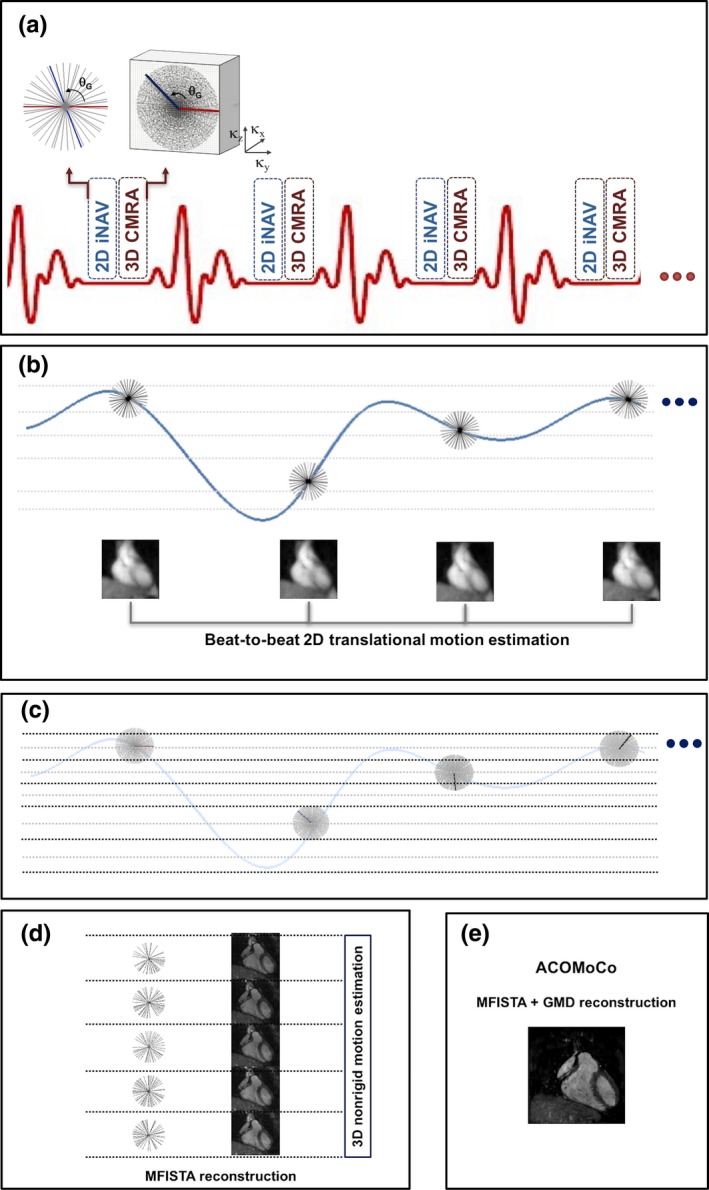
Proposed acquisition and reconstruction framework: (a) a golden angle radial 2D image navigator (iNAV) is acquired at every heartbeat interleaved with a segment of an undersampled 3D CMRA scan, performed using a variable‐density radial Cartesian trajectory; (b) the iNAVs are used to estimate the beat‐to‐beat 2D translational motion and to derive the superior–inferior respiratory signal; (c) the 3D CMRA data are separated into respiratory bins and corrected for 2D translational motion; (d) bin images are reconstructed using MFISTA with total variation regularization and registered to estimate the bin‐to‐bin 3D nonrigid motion; (e) the nonrigid motion fields are incorporated into the proposed ACOMoCo reconstruction, which combines a TV‐regularized version of MFISTA with motion compensation using the general matrix description (GMD). [Color figure can be viewed at wileyonlinelibrary.com]

Data were acquired using an interleaved scanning framework.^32^ For each cardiac cycle, a highly undersampled 2D image navigator (iNAV) was acquired using a golden radial (GR) k‐space trajectory.^33^, ^34^ Each iNAV was interleaved with a segment of the undersampled 3D CMRA scan. The undersampled 3D CMRA data were acquired using a variable‐density Cartesian trajectory with radial profile order in the k_y_–k_z_ plane, where the radial‐like spokes were separated by the golden angle θ_G_ = 111.25° [Fig. 1(a)]. The so‐called VD‐CAPR trajectory was implemented using a strategy similar to the variable‐density spiral trajectory described in Ref. 35.

### 2.B. Image reconstruction and motion correction

Motion estimation and correction was performed in two steps: (a) 2D iNAVs are used to estimate the beat‐to‐beat translational motion [Fig. 1(b)], (b) 3D bin‐to‐bin nonrigid motion is estimated from respiratory‐resolved images obtained from CS reconstructions of undersampled 3D CMRA data [Figs. 1(c) and 1(d)]. Finally, the proposed approach was used to produce a motion‐compensated reconstruction from free‐breathing undersampled CMRA data [Fig. 1(e)]. This is achieved by incorporating nonrigid motion correction in a CS reconstruction algorithm using the general matrix description (GMD) approach.^36^

#### 2.B.1. Beat‐To‐Beat translational motion

The iNAVs were reconstructed using a gridding method^37^ combined with an iterative density compensation scheme.^38^ This provided a set of low‐resolution images with high temporal resolution, which were used to estimate the beat‐to‐beat 2D translational motion (SI and right‐left: RL). This was achieved by selecting a region of interest around the heart^34^ and estimating the translational motion via image registration.^39^ Respiratory outliers due to deep breaths were removed. Typically, values greater than two standard deviations were considered outliers. The 3D CMRA data were separated into five equally populated respiratory bins according to the SI motion information. The k‐space data within each bin were corrected for 2D translational motion by applying the corresponding phase shift correction.^19^

#### 2.B.2. Respiratory‐resolved bin reconstruction from undersampled CMRA data

The forward problem for each bin *b* is given by: $k_{b}=E_{b}u_{b}+\varepsilon ,$ where the operator $E_{b}=A_{b}\;F\;S$ relates the (unknown) bin image **u**_*b*_ to the (undersampled) measured k‐space data **k**_*b*_ of the corresponding respiratory bin, corrupted by noise **ɛ**. More specifically, **E**_*b*_ is an encoding matrix that incorporates the sampling matrix **A**_*b*_ for bin *b*, Fourier transform F, and coil sensitivities S.

Each bin image **u**_*b*_ is reconstructed by solving the following minimization problem:

(1) $${\hat{u}}_{b}=\arg\min_{u_{b}}\left\{\frac{1}{2}{∥W_{b}\left(E_{b}u_{b}-k_{b}\right)∥}^{2}+\lambda \Psi_{\mathrm{TV}}\left(u_{b}\right)\right\},$$

where the first term is the L2‐norm of the residual or data consistency for each bin, *λ* is the regularization parameter and $\Psi_{\mathrm{TV}}\left(u_{b}\right)$ is the 3D total variation (TV) regularization function given by: $\Psi_{\mathrm{TV}}\left(u_{b}\right)=-3\;\sum_{i}\sqrt{{\left[D_{i}^{x}\left(u_{b}\right)\right]}^{2}+{\left[D_{i}^{y}\left(u_{b}\right)\right]}^{2}+{\left[D_{i}^{z}\left(u_{b}\right)\right]}^{2}\;},$ where *D* ^*x*^, *D* ^*y*^, and *D* ^*z*^ represent the first‐order finite differences of **u**_*b*_ at voxel *i* in the *x*,* y*, and *z* direction, respectively. TV regularization is required because 3D CMRA is undersampled, and thus, each bin is highly undersampled. This is in contrast to the work by Cruz et al.,^19^ where 3D CMRA was fully sampled, and hence, much lower undersampling factors were expected for each bin. Moreover, to reduce aliasing artifacts resulting from the high undersampling factors and ensure good‐quality reconstructions for each bin, here we use a variable‐density trajectory (VD‐CAPR). The VD‐CAPR trajectory combines some of the advantages of radial and Cartesian trajectories. The central region of k‐space is oversampled, thus offering reduced sensitivity to motion without the disadvantages of radial sampling, such as lower signal‐to‐noise ratio (SNR), sensitivity to off‐resonance, and computational complexity of the reconstruction.

The data fidelity term is weighted according to the SI respiratory distance of a k‐space point relative to the center of the bin *b* being reconstructed using soft‐gating,^29^, ^40^ where **W**_*b*_ is a matrix that contains weights for each k‐space position. Therefore, data points that fall within the same respiratory position (or bin) are considered to be motion free (weights close to 1), whereas data points from far distant bins have weights close to zero. An exponential decay weighting was used as described in Ref. 40. Soft‐gating is particularly suitable to reconstruct highly undersampled datasets while only introducing minor motion blurring.

The solution to the minimization problem in Eq. (1) was obtained using the monotone version of the fast iterative shrinkage‐thresholding algorithm (MFISTA).^41^ This method guarantees a monotonic decrease of the objective function and fast rate of convergence. The iterative scheme proposed by Chambolle^42^ was used to find the solution of the TV denoising subproblem, which is necessary to solve at each iteration.

#### 2.B.3. Bin‐to‐Bin nonrigid motion

The reconstructed high‐resolution bin images were used to estimate the 3D nonrigid respiratory motion via image registration using free‐form deformations based on B‐splines,^43^, ^44^ where the end‐expiration bin was used as reference.

Residual inter‐bin motion is corrected using the following compressed sensing motion‐corrected reconstruction (ACOMoCo):

(2) $$\hat{v}=\arg\min{}_{v}\left\{\frac{1}{2}{∥\mathrm{Ev}\text{‐}k∥}^{2}+\lambda \Psi_{\mathrm{TV}}\left(v\right)\right\},$$

where $\hat{v}$ is the motion‐corrected CMRA image reconstructed from undersampled k‐space data **k** and $E=\sum_{b}A_{b}F\;S\;U_{b}$ follows the GMD formalism introduced by Batchelor et al.,^36^ where **A**_*b*_ is the sampling matrix for bin *b* and **U**_*b*_ are the nonrigid motion fields estimated for each bin. Unlike the work in Ref. 19 where the 3D CMRA was fully sampled, 3D TV regularization, $\Psi_{\mathrm{TV}}\left(v\right)$, is necessary here to reduce undersampling artifacts in the motion‐corrected image. The solution to the minimization problem in Eq. (2) was obtained using MFISTA.

### 2.C. In vivo experiments

Ten healthy subjects were scanned on a 1.5 T Philips Ingenia scanner (Philips Healthcare, Best, The Netherlands) using a 12‐channel posterior coil and 16‐channel anterior coil. The study was approved by the Institutional Review Board and written informed consent was obtained from all subjects before the scan.

As described before, one k‐space segment of the 3D not respiratory‐gated CMRA scan and one 2D GR iNAV were acquired per cardiac cycle using electrocardiography (ECG)‐triggering. The 3D CMRA acquisition was performed using a balanced steady‐state free precession sequence with the following parameters: isotropic resolution = 1.2 × 1.2 × 1.2 mm^3^; field of view = 300 × 300 × 100 mm^3^; repetition time (TR)/echo time (TE) = 5/2.5 ms; flip angle = 90°; T2 preparation (50 ms); fat saturation prepulse; subject specific mid‐diastolic trigger delay and acquisition window (116–120 ms corresponding to 22–24 readouts per segment); and a low‐high VD‐CAPR acquisition 3× undersampled in the k_y_–k_z_ plane. Additionally, a conventional twofold Cartesian SENSE‐accelerated^45^ scan with diaphragmatic respiratory gating and tracking (5 mm acceptance window and tracking scaling factor of 0.6) was performed for comparison. Normally, a fully sampled acquisition would be preferred. However, fully sampled high‐resolution isotropic acquisitions are infeasible, due to the extremely long scan times required. For the 2D GR iNAV acquisition, a spoiled gradient echo sequence was used with the following parameters: 4 × 4 mm^2^ in‐plane resolution; slice thickness = 25 mm; field of view = 300 × 300 mm^2^; TR/TE = 1.9/0.78 ms; flip angle = 5°; acquisition window = 46.1 ms with 24 angular profiles per cardiac cycle.

For each undersampled CMRA dataset, images were obtained using a nonmotion‐corrected iterative SENSE (NMC) reconstruction^46^ and the proposed ACOMoCo approach. Additionally, a SENSE reconstruction was obtained from each reference dataset, i.e., navigator‐gated and tracked acquisition. In the proposed ACOMoCo approach, data were separated into five respiratory bins with equal amount of data, which corresponded to an undersampling factor of ~15× for each bin. MFISTA was used to solve Eqs. (1)) and (2), and the regularization parameter *λ* was selected empirically. For reconstructions of binned data [Eq. (1)], *λ* was set to 0.008 for all bins and for the final reconstruction [Eq. (2)], *λ* was set to 0.005. For each MFISTA outer iteration, five additional inner iterations were performed to solve the TV subproblem. The stopping criteria was (a) the maximum number of outer iterations, which was set to 20, and (b) the relative difference between consecutive cost function values lower than a tolerance, set to 10^−4^.

The proposed framework required five translation‐corrected soft‐gated iterative MFISTA bin reconstructions, taking approximately 1520 s. Then, nonrigid motion registration was performed (~142 s) followed by the final ACOMoCo reconstruction (~963 s). Hence, the total reconstruction time was approximately 2625 s. All reconstructions were performed offline using MATLAB (Mathworks, Natick, MA, USA) on a Linux PC with 32 Intel Xeon E5‐2680 CPUs @ 2.70 GHz and 198 GB memory.

All reconstructions were reformatted using “Soap‐Bubble”^47^ to enable simultaneous display of the right coronary artery (RCA) and left anterior descending artery (LAD). Using the same software, the quality of the reconstructions was quantified in terms of RCA and LAD vessel length and sharpness. Vessel length of a tracked vessel was measured from a user‐specified pathway along individual coronary segments. Vessel sharpness was calculated by taking the maximum gradient normal to the vessel of interest normalized to the signal intensity of the vessel centerline. Vessel sharpness was measured in the proximal 4 cm and full length of both RCA and LAD. Quantitative differences between the navigator‐gated and ACOMoCo methods were tested for statistical significance using a paired t‐test (*P* < 0.05).

## 3 Results

**Figure 2:**
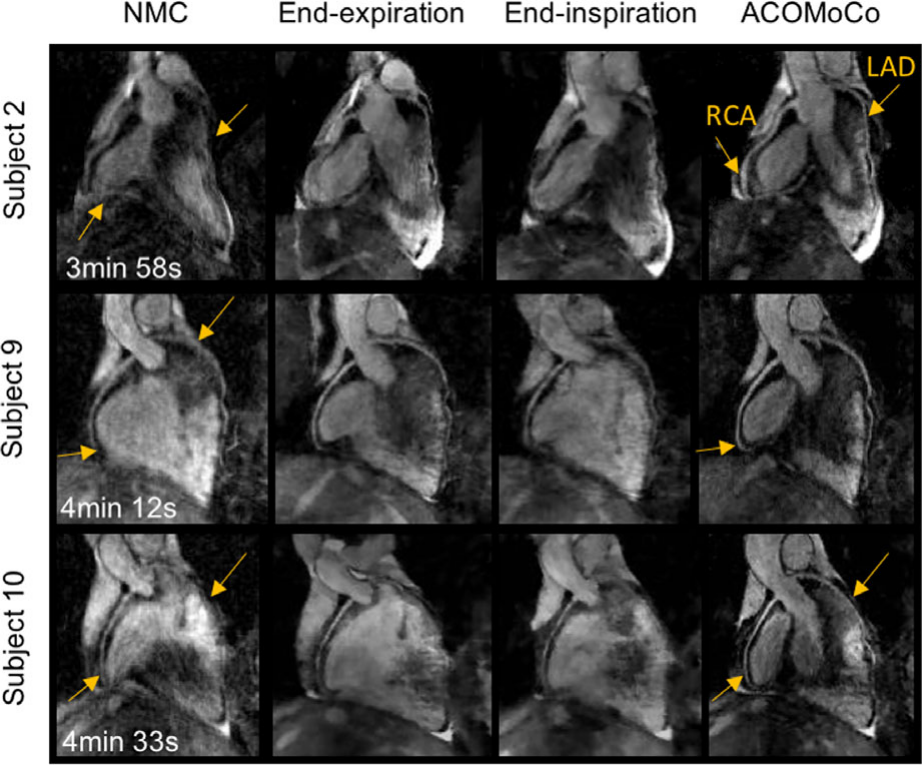
Reformatted images showing the right coronary artery (RCA) and left anterior descending coronary artery (LAD) for three representative subjects. Reconstructions were obtained from not respiratory gated 3× undersampled CMRA data using (left) nonmotion‐corrected parallel imaging reconstruction (NMC) and (right) proposed method. Reconstructions of 15× undersampled end‐expiration and end‐inspiration bins were obtained using MFISTA (middle). Significant motion blurring is observed in the NMC images (arrows). Respiratory binning greatly improves the quality of the images by reducing the amount of motion in each bin, but it increases the noise level and remaining undersampling artifacts are observed. The proposed method significantly improves the visibility and sharpness of both coronaries (arrows). Total acquisition times are indicated for each subject. The corresponding twofold accelerated navigator‐gated acquisition times are (top to bottom) 17 min 24 s, 18 min 15 s, and 17 min 33 s. [Color figure can be viewed at wileyonlinelibrary.com]

**Figure 3:**
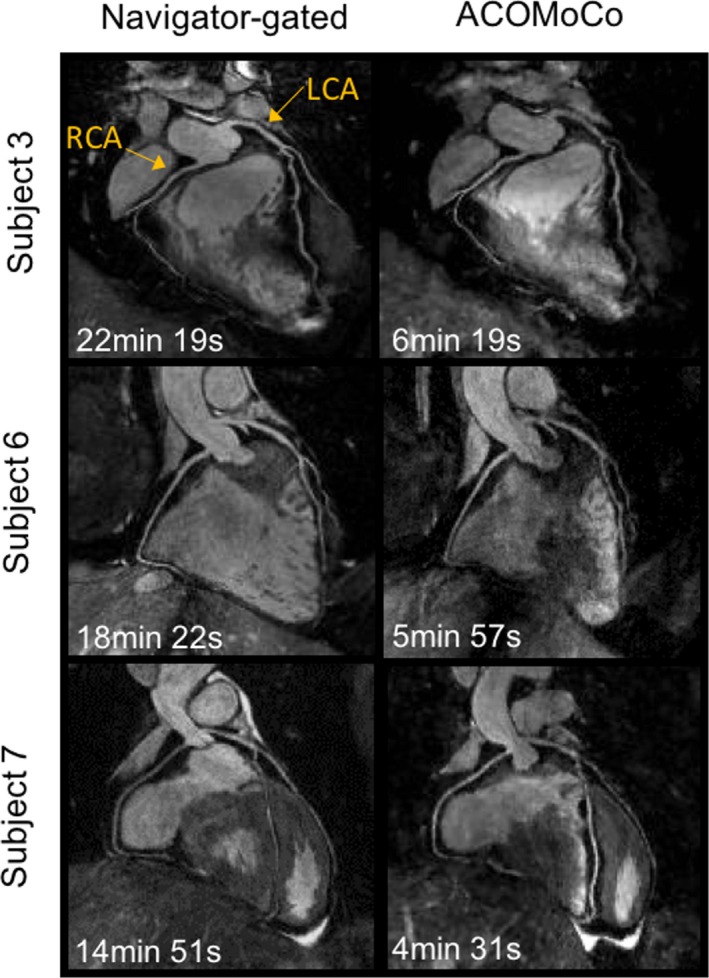
Reformatted images along the right coronary artery (RCA) and left anterior descending coronary artery (LAD) for three representative subjects: (left) twofold SENSE‐accelerated navigator‐gated and tracked acquisition with 5‐mm gating window and 0.6 scaling factor; (right) free‐breathing threefold undersampled VD‐CAPR acquisition reconstructed using the proposed ACOMoCo approach. The proposed method provides images of comparable quality to navigator‐gated scans. However, slightly decreased quality is observed in the distal segment of the RCA. Total acquisition times are indicated in each subfigure. [Color figure can be viewed at wileyonlinelibrary.com]

Figure 2 shows representative whole‐heart reformatted images displaying the RCA and LAD, for three subjects, obtained from reconstructions of 3× undersampled not respiratory gated VD‐CAPR data using the NMC and ACOMoCo methods. Additionally, end‐expiration and end‐inspiration respiratory bin reconstructions are displayed. The maximum and average SI respiratory motion amplitudes were 31.94 mm and 11.27 ± 2.97 mm, respectively. The overall estimated SI, AP (anterior–posterior), and RL contributions were 76.54% ± 13.53%, 11.29% ± 4.27%, and 12.16% ± 3.56%, respectively. All respiratory bin images can be seen in Figure S1. In addition, a thorough analysis of the motion fields is provided in the supporting information (Section S2). Significant undersampling and motion blurring artifacts can be observed in both coronaries when nonmotion compensation and parallel imaging reconstruction is used. Respiratory binning efficiently reduces motion, particularly for end‐expiration bins, which usually contains less motion than end‐inspiration bins (see supporting information Section S1 for further details). Hence, reconstructed end‐expiration bin images have in general higher image quality. However, the resulting high undersampling factors inevitably lead to stronger undersampling artifacts and signal‐to‐noise ratio loss. For all subjects, the proximal segment of the RCA appears much sharper when the proposed motion‐corrected CS reconstruction method is used. Moreover, the proposed method allows visualization of the distal segment of the RCA (arrows). For subjects 2 and 10, the LAD was extremely blurred in the NMC images, resulting in limited visibility of the vessel structure. These challenging cases were improved using the proposed ACOMoCo method (arrows). Overall, reconstruction artifacts and blurring are minimized with the proposed approach, leading to improved coronary visibility. The gating efficiency of the 2×‐accelerated 5‐mm navigator‐gated and tracked reference scan was 43.1% ± 10.2% across all subjects, whereas for the proposed ACOMoCo approach, it was 97.0% ± 2.6%. The total acceleration factor (undersampling and gating efficiency) obtained with the proposed method compared to the reference scan ranged from 2.4 to 4.4, with a mean acceleration across subjects of 3.5 ± 0.7. The average acquisition time was reduced from 17 ± 4 min to 5 ± 1 min. Figure 3 shows coronal images reformatted from navigator‐gated and ACOMoCo images to visualize the RCA and LAD of three subjects. For subject 7, it is also possible to display the circumflex coronary in the same reformatting. Similar image quality was achieved with the proposed method and reference navigator‐gated approach. However, a slight reduced visibility of the distal segment of the RCA can be observed for subjects 6 and 7 in the ACOMoCo reconstructions.

**Figure 4:**
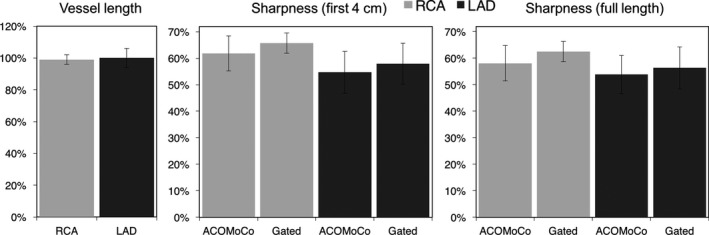
Image metrics for 10 subjects obtained from images reconstructed with the proposed ACOMoCo method and navigator‐gated approach: (left) vessel length, (middle) vessel sharpness for the first 4 cm (right) and full length of the right coronary artery (RCA) and left anterior descending coronary artery (LAD).

Quantitative image quality was assessed in terms of vessel sharpness and length, as shown in Figure 4. The measured vessel length (normalized to the navigator‐gated scan vessel length) for the RCA and LAD, respectively, was 99% ± 3% and 100% ± 6% for ACOMoCo. There were no significant differences in vessel length between the navigator‐gated and the proposed approaches for both coronaries. There were no significant differences in vessel sharpness between the proposed method and reference case when analyzing the proximal segment and full length of both coronaries. However, the proposed approach provides slightly lower vessel sharpness for both RCA and LAD.

## 4 Discussion

A novel nonrigid motion‐compensated reconstruction method for free‐breathing undersampled 3D CMRA has been proposed. The method combines compressed sensing and nonrigid motion correction in a unified TV‐regularized reconstruction. Motion correction was achieved in two steps: (a) 2D iNAVs are used to estimate beat‐to‐beat translational motion, which is used to group data into respiratory bins and correct for intra‐bin 2D translational motion in k‐space; (b) these highly undersampled bins are reconstructed, by solving a TV‐based CS problem using MFISTA, and used to estimate 3D bin‐to‐bin nonrigid motion. The estimated nonrigid motion fields are incorporated into a TV‐regularized reconstruction using the general matrix formalism.

Here, the proposed method obtains a nonrigid motion‐compensated CS reconstruction from a 3× undersampled 3D CMRA dataset. Our approach leads to a total acceleration of ~8× in comparison to a conventional fully sampled navigator‐gated acquisition with 43% scan efficiency (average gating efficiency across all subjects in this study), as an acquisition speed up of ~3× is achieved due to undersampling and of ~2.7× due to respiratory motion correction, which enables ~100% scan efficiency. If respiratory outliers are preserved, 100% scan efficiency is reached. In addition, note that an elliptical shutter was applied for further acceleration, which was not accounted for in the net acceleration factor. The achieved scan acceleration was facilitated by employing a variable‐density Cartesian trajectory and incorporating TV regularization to reconstruct both the highly undersampled bin images (required for nonrigid motion estimation) and the final undersampled and motion‐corrected CMRA image. In contrast, the work of Cruz et al.,^19^ that performs a nonrigid motion‐compensated reconstruction from a fully sampled 3D CMRA acquisition, achieves ~2× total acceleration which is due to motion correction only.

Images were reconstructed using NMC and the proposed ACOMoCo method from threefold undersampled datasets, acquired using a VD‐CAPR trajectory with isotropic resolution. These approaches were compared with a conventional twofold Cartesian SENSE‐accelerated acquisition diaphragmatic navigator‐gated acquisition with 5‐mm gating window and tracking factor of 0.6. As expected, the proposed approach substantially reduced respiratory motion and undersampling artifacts, which were visible in NMC images. There were no significant differences in vessel sharpness and length between the proposed method and navigator‐gated approach, for both RCA and LAD. The proposed method provides comparable image quality to the conventional navigator‐gated images, while reducing the scan time by a factor of ~4. Additionally, ACOMoCo provides more predictable scan times.

Vessel sharpness scores were slightly higher for the navigator‐gated approach than ACOMoCo. This could be due to residual cardiac or respiratory motion. Cardiac motion could be reduced by using a shorter mid‐diastolic acquisition window (~80 ms), particularly for subjects with higher heart rates, at the expense of longer scan times. Moreover, arrhythmia rejection techniques can be included to account for variations in heart rate and reject very irregular R‐R intervals.^48^, ^49^ Intra‐bin residual translational motion may be addressed by acquiring 3D iNAVs, using VD‐CAPR or spiral phyllotaxis^50^ sampling, to estimate beat‐to‐beat 3D translational motion. Thus, additionally allowing for intra‐bin translational motion correction in the AP direction. A preliminary study showed that 3D iNAVs with spatial resolution of 5 × 10 × 10 mm^3^, acquired in 81 ms, can be used to efficiently estimate 3D motion.^51^ Furthermore, residual nonrigid motion can be reduced by increasing the number of bins. However, undersampling artifacts will increase, which could compromise motion estimation accuracy. In this case, a regularization term that exploits temporal sparsity, i.e., along the respiratory dimension,^8^ could be added to guarantee that bin reconstructions have sufficient quality for reliable motion estimation. This strategy can be particularly useful in clinical settings, for imaging patients with highly irregular breathing patterns. However, reconstruction times increase with the number of bins. Hence, parallel implementation of the proposed method on a graphics processing unit (GPU) is needed before proceeding to clinical applications.

As mentioned previously, the VD‐CAPR trajectory combines advantages of radial and Cartesian trajectories. However, like radial sampling, it may suffer from slight blurring, which can lead to lower vessel sharpness. Nevertheless, the VD‐CAPR is particularly suitable for the respiratory binning step. Other Cartesian trajectories, such as Cartesian acquisition with spiral order profile (CASPR)^18^ or Cartesian acquisition with projection‐reconstruction‐like (CAPR),^52^ may achieve higher image sharpness when motion is not present. However, these trajectories may not provide respiratory bin images with sufficient quality for accurate nonrigid motion estimation, particularly when motion is estimated from undersampled CMRA data. This is because the region where most information is concentrated, i.e., the center of k‐space, may not be sufficiently sampled for each bin.

Scan acceleration leads to a reduced SNR, which combined with SNR falling off rapidly with depth leads to reduced visibility of distal artery segments. The images obtained with the proposed method could be further improved by using a spatially varying regularization parameter, to compensate for spatial variations in SNR. Moreover, automatic selection of an optimal regularization parameter could also improve the quality of reconstructions.

Methods that combine CS and motion compensation techniques have been applied to free‐breathing CMRA to achieve a shorter and more predictable scan time. Forman et al.^29^ proposed a 1D self‐navigated CMRA strategy combined with soft‐gated CS reconstruction, to reduce acquisition time and compensate for SI respiratory motion. Moghari et al.^30^ accelerated acquisitions using CS and acquired 3D iNAVs to estimate 3D translational motion, which was used to correct the CMRA k‐space data for respiratory motion. Data were partially acquired using a conventional navigator‐gated acquisition, leading to a less predictable scan time. Similar to these methods, the proposed ACOMoCo approach aims to accelerate acquisitions by combining CS with motion compensation. However, ACOMoCo achieves high scan efficiency and corrects for more complex nonrigid motion, but at the expense of higher computational cost. The computational complexity of the bin reconstructions and ACOMoCo is 2N_s_N_b_ *O*(2N logN), where N_s_ and N_b_ are the number of coil channels and bins, respectively, and N is the number of samples. At present, a limitation of this technique is the long reconstruction time (~44 min). However, it could be substantially reduced using a GPU‐based implementation.

Whole‐heart CMRA with sub‐mm isotropic resolution was achieved using CS techniques to accelerate navigator‐gated acquisitions.^26^, ^27^ Future studies will aim to further develop the acquisition to achieve sub‐mm resolution, reduce the computational complexity and speed up the reconstruction, and will evaluate the proposed technique in patients.

## 5 Conclusions

In summary, highly accelerated 3D Cartesian CMRA is achieved by undersampling the acquisition using a VD‐CAPR trajectory and performing nonrigid respiratory motion correction directly in the CS reconstruction. This approach enables free‐breathing 1.2 mm isotropic CMRA acquisitions in ~5 min. The proposed method estimates 2D beat‐to‐beat translational motion from iNAVs and 3D nonrigid motion is estimated directly from undersampled 3D CMRA data. Accurate nonrigid motion estimation is facilitated by using a VD‐CAPR trajectory and TV‐regularized reconstruction, which allows high‐quality respiratory bin reconstructions. Nonrigid motion is incorporated into the final reconstruction, which combines CS and general matrix description, enabling CMRA acquisitions up to about four times faster than conventional twofold accelerated navigator‐gated and tracked scans with comparable quality and more predictable scan time.

## Supporting information


**Data S1.** Technical Note: Accelerated nonrigid motion compensated isotropic 3D coronary MR angiography
