# Supplementary material for: Technical note: Accelerated nonrigid motion‐compensated isotropic 3D coronary MR angiography
Source: Med Phys. 2017 Dec 12;45(1):214–22. doi: 10.1002/mp.12663 (PMC5814733; doi:10.1002/mp.12663)
Supplement: Supplementary file 1 — Data S1. Technical Note: Accelerated nonrigid motion compensated isotropic 3D coronary MR angiography [file MP-45-214-s001.pdf]

Supplemental material

Technical Note: Accelerated nonrigid motion-compensated isotropic 3D coronary MR angiography

T Correia*, G Cruz, T Schneider, R M Botnar, Claudia Prieto
*E-mail: teresa.correia@kcl.ac.uk

S1. Respiratory-resolved reconstructions

The proposed ACOMoCo method requires the reconstruction of respiratory-resolved images. In our study, the 3D CMRA data was separated into five respiratory bins. The bins were numbered in ascending order from end-inspiration to end-expiration, i.e., bin 1 corresponds to end-inspiration and bin 5 to end-expiration. In ascending bin number order, the bin widths were 8.2 mm ± 1.5 mm, 6.6 mm ± 2.9 mm, 4.3 mm ± 1.3 mm, 3.2 ± 1.6 mm and 4.8 mm ± 1.4 mm. Therefore, more residual respiratory motion was present in near end-inspiration bins. Figure S1 shows the nonmotion-corrected reconstruction, the five bin reconstructions and ACOMoCo images for three representative subjects. Respiratory binning reduces respiratory motion very efficiently, but it results in highly undersampled bins, which inevitably leads to higher levels of noise and remaining undersampling artifacts are observed. The proposed ACOMoCo method significantly improves the visibility of both coronaries, by using all the acquired 3D CMRA data and correcting for nonrigid motion. Even though respiratory-induced cardiac motion is dominated by superior-inferior motion, there are important motion components along other directions. In the following section, a thorough analysis of the motion fields is performed to show the proportions of the different respiratory motion components.

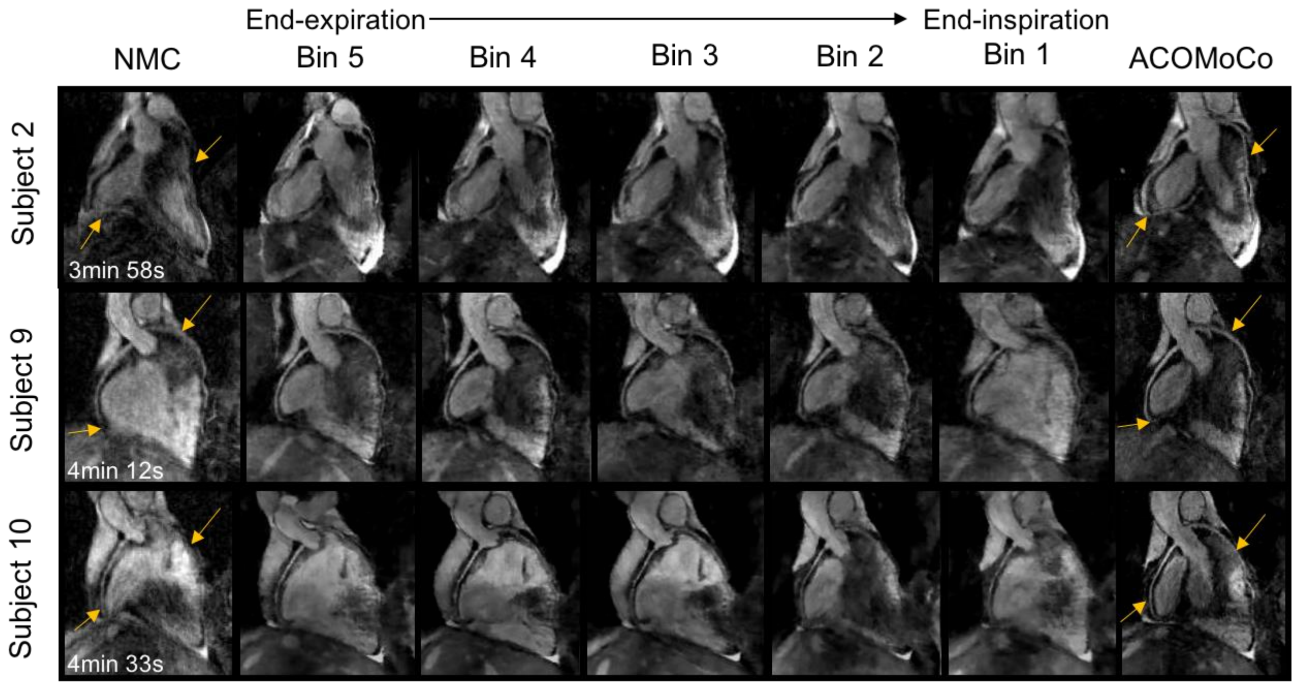

Fig. S1: Reformatted images showing the right coronary artery (RCA) and left anterior descending coronary artery (LAD) for three representative subjects. Reconstructions were obtained from not respiratory gated 3x undersampled CMRA data using (left) nonmotion-corrected parallel imaging reconstruction (NMC) and (right) proposed ACOMoCo method. Reconstructions of 15x undersampled respiratory bins were obtained using MFISTA (five middle images). Significant motion blurring is observed in the NMC images (arrows). Respiratory binning greatly improves the quality of the images by reducing the amount of motion included in each bin. However, it increases the noise level and degree of undersampling, and hence, the undersampling artifacts. The proposed method significantly improves the visibility and sharpness of both coronaries (arrows). Total acquisition times are indicated for each subject. The corresponding two-fold accelerated navigator-gated acquisition times are (top to bottom) 17min 24s, 18min 15s and 17min 33s.

S2. Motion field analysis

Nonrigid image registration was performed using the NiftyReg software package [44], which is based on free-form deformations [43]. It uses an objective function that incorporates a measure of similarity (normalized mutual information) and a regularization term (bending energy). The weight of the bending energy penalty term was set to 0.0001 and the grid spacing was set to 8 voxels. The remaining parameters were set to their default values.

The nonrigid motion compensation framework required five respiratory-resolved bin reconstructions. The end-expiration bin was used as the reference image and four motion fields were obtained. For each motion field an analysis was performed to identify the proportions of superior-inferior (SI), anterior-posterior (AP) and right-left (RL) contributions in each motion vector. First, the heart was divided into 27 regions of interest (ROIs) as shown in Figure S2. Then, for each subject, the directional components normalized to the overall maximum were averaged within each ROI. Finally, the mean and standard deviation over all subjects was calculated. Figure S3 shows the estimated SI, AP and RL contributions for all the ROIs, normalized to the maximum stacked value, for the motion field between the reference image (bin 5) and the image corresponding to bin 1. The estimated directional contributions corresponding to bins 2-4 are shown in Figures S4-S6, respectively. Figure S7 shows the average SI, AP, RL contributions over the four motion fields. The overall estimated SI, AP and RL contributions were 76.54% ± 13.53%, 11.29% ± 4.27% and 12.16% ± 3.56%, respectively. As expected, the SI contribution to respiratory-induced cardiac motion is larger than the AP and RL motion.

Figures S3-S7 show that SI motion is generally more significant in anterior regions of the heart. However, AP contributions to motion are larger in posterior/middle regions. In addition, the left side of the base of the heart exhibits the largest SI amplitude. Otherwise, in general the regions at mid-distance between the base and apex of the heart have the largest SI motion. The differences between the mean values obtained for each ROI become less evident the closer the respiratory bin images are to the reference image. However, the standard deviations increase, showing that there is higher inter-subject variability. Figure S8 displays the motion fields obtained for three representative subjects, which shows how greatly the amplitude and direction of motion varies from subject to subject.

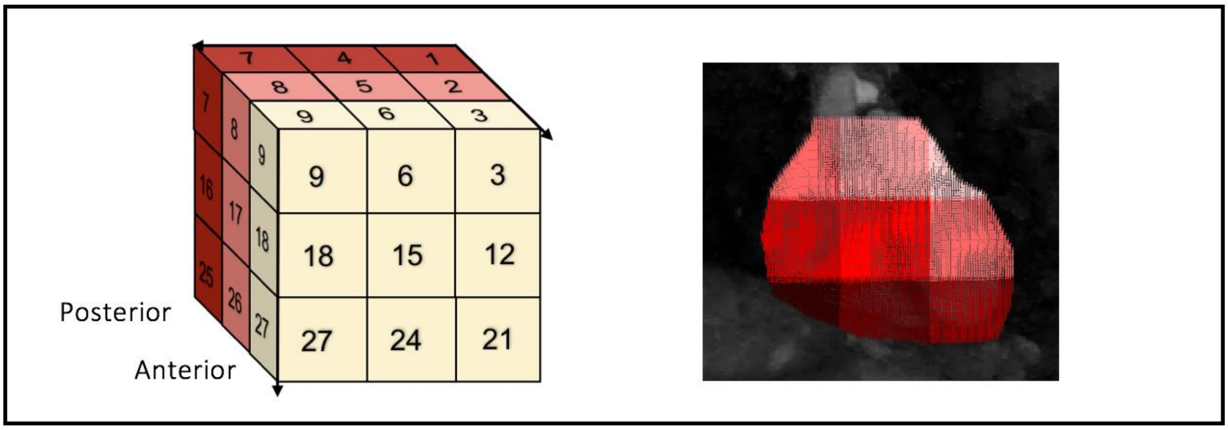

Fig. S2: For the motion fields analysis (left) the heart was divided into 27 regions. (right) Coronal slice showing 9 regions of interest.

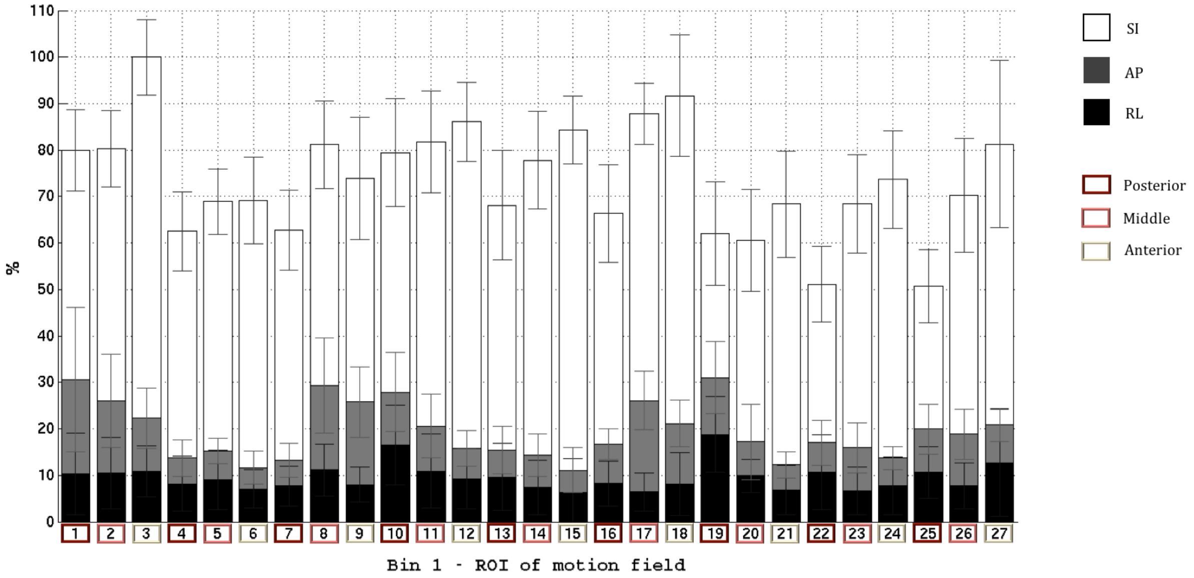

Fig. S3: Superior-inferior (SI), anterior-posterior (AP) and right-left (RL) contributions within each ROI of the estimated motion field between reference bin 5 and bin 1.

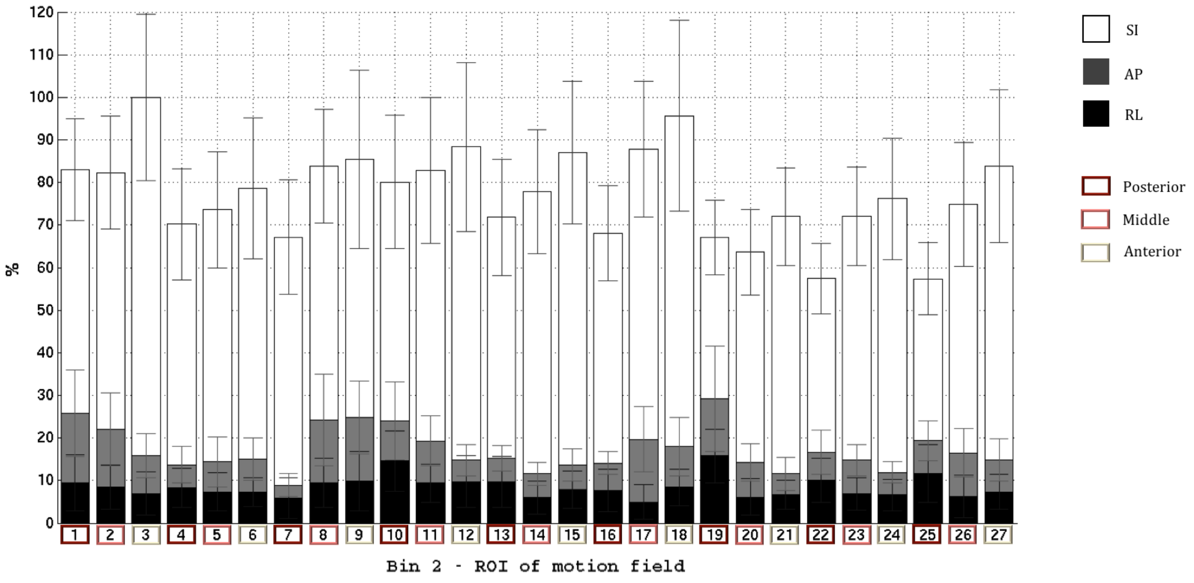

Fig. S4: Superior-inferior (SI), anterior-posterior (AP) and right-left (RL) contributions within each ROI of the estimated motion field between reference bin 5 and bin 2.

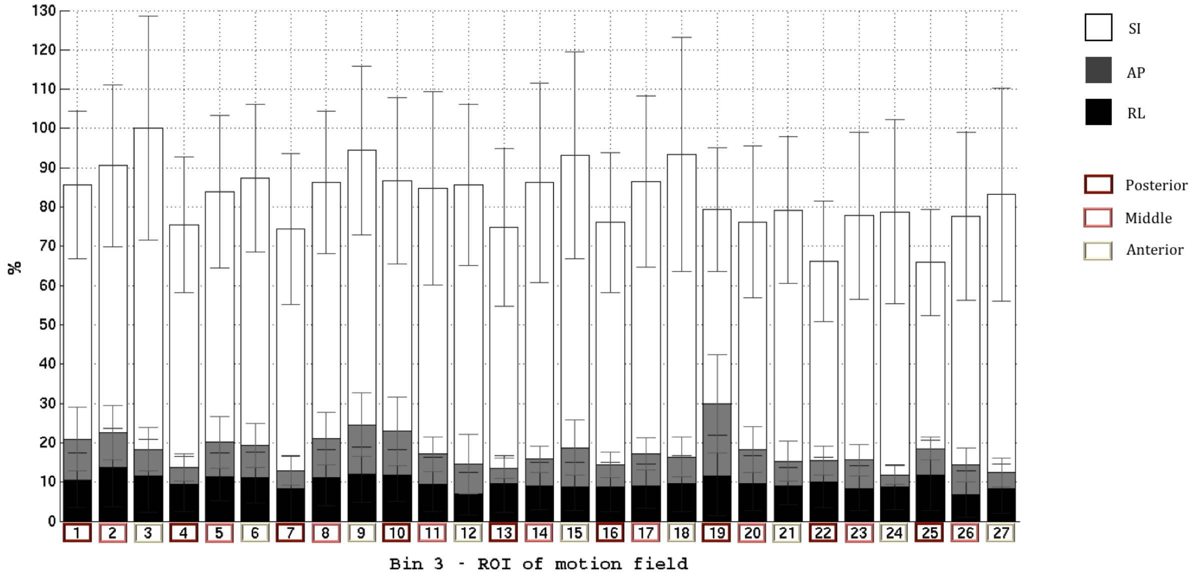

Fig. S5: Superior-inferior (SI), anterior-posterior (AP) and right-left (RL) contributions within each ROI of the estimated motion field between reference bin 5 and bin 3.

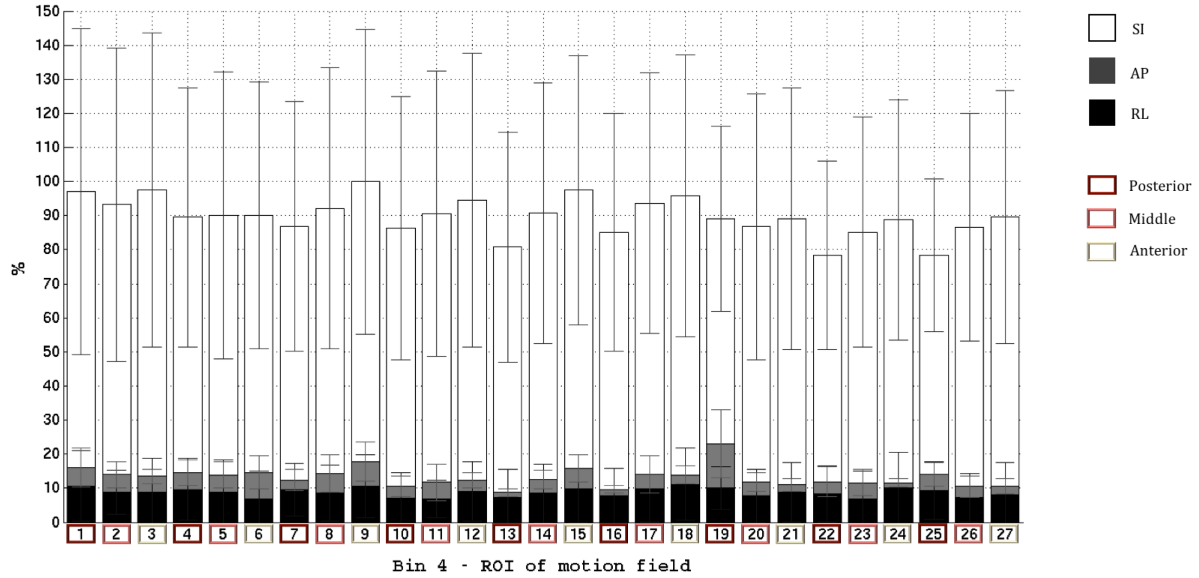

Fig. S6: Superior-inferior (SI), anterior-posterior (AP) and right-left (RL) contributions within each ROI of the estimated motion field between reference bin 5 and bin 4.

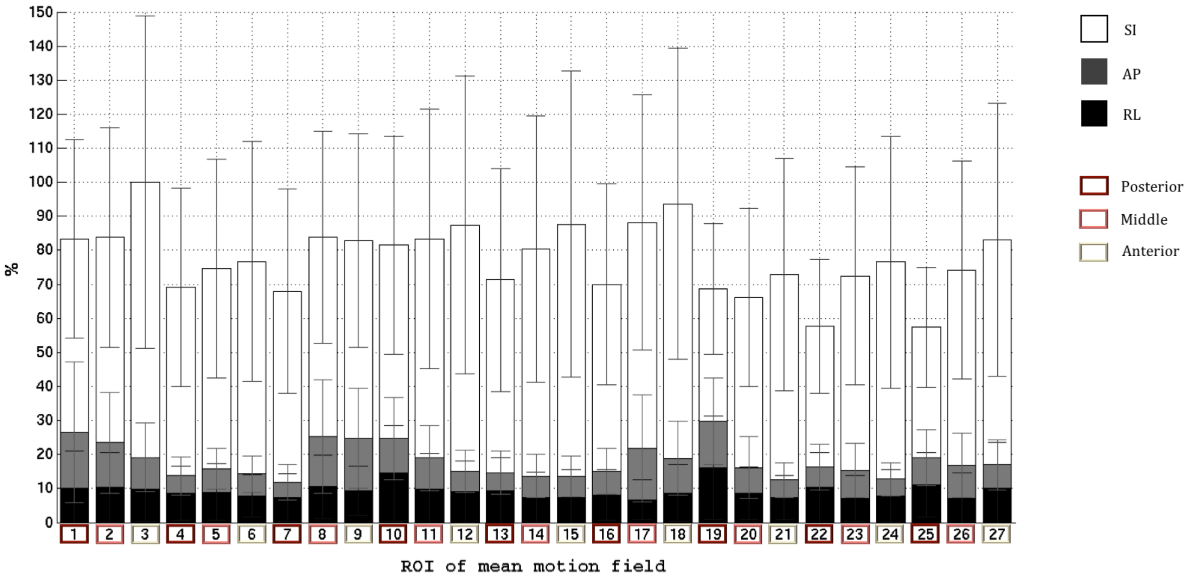

Fig. S7: Superior-inferior (SI), anterior-posterior (AP) and right-left (RL) contributions within each ROI of the mean motion field.

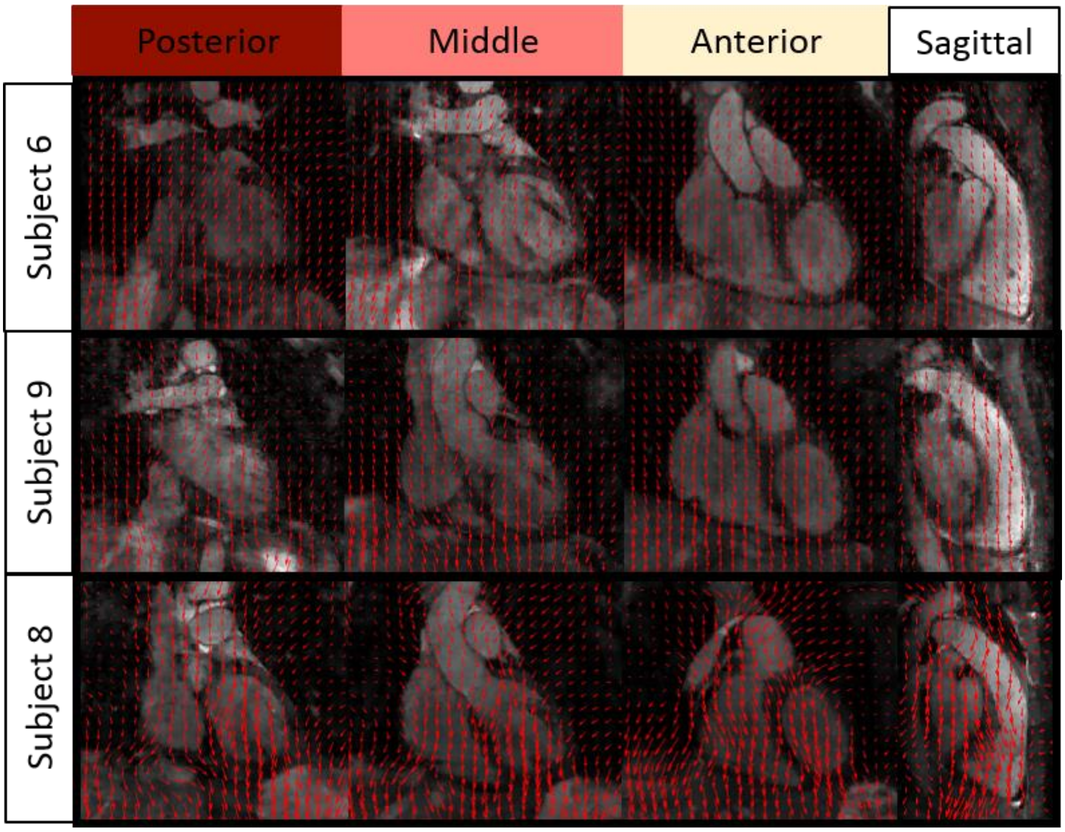

Fig. S8: Motion fields obtained between the end-expiration image and the end-inspiration image for three subjects, which exhibit (top to bottom) small, medium and large motion amplitude. A sagittal slice is displayed together with three coronal slices, which correspond to posterior, middle and anterior sections.
